# Immunomodulatory Effects of Cell Therapy after Myocardial Infarction

**DOI:** 10.33696/immunology.3.082

**Published:** 2021

**Authors:** Joseph B Moore, Marcin Wysoczynski

**Affiliations:** Diabetes and Obesity Center, University of Louisville School of Medicine, Louisville, KY, USA

Myocardial infarction (MI) due to coronary artery stenosis compromises vascular endothelial integrity and increases vascular permeability [1,2]. Concurrently, ensuing myocardial tissue death and necrosis results in the release of danger associated molecular patterns (DAMPs), cytokines, chemokines, bioactive lipids, as well as activation of the complement cascade [1-3]. Collectively, these events direct a pronounced and immediate immune response, which includes the recruitment of peripheral blood leukocytes to the site of injury [2,3]. These infiltrating neutrophils are primarily responsible for the clearance of necrotic tissue and cellular debris in ischemic regions via their release of a host of proteolytic enzymes/proteases. While this constitutes a necessary early step in the myocardial repair process at the site of injury, neutrophil-derived reactive oxygen species (ROS) and pro-inflammatory cytokines/chemokines can contribute to collateral damage of surviving myocardium and amplify tissue injury [3,4]. Nevertheless, neutrophils are imperative for proper infarct healing as their depletion prior to MI leads to a dysregulated immune response, excessive scarring, and impaired ventricular function [5]. Within days of an MI, neutrophils undergo cell death and disappear from infarcted tissue [3,4]. Recruitment of neutrophils is followed by two waves of monocyte infiltration. First, early recruitment of Ly6C^High^ monocytes expressing pro-inflammatory cytokines, and second, infiltration of Ly6C^Low^ monocytes with pro-resolving and pro-reparative function [3,6,7]. Ly6C^High^ monocyte migration is driven by the presence of tissue CCL2 chemokine gradients and their interaction with their cognate receptor, CCR2 [8]—a group of monocytes that are principally sourced from bone marrow and spleen. Subsequently, these monocytes differentiate into Ly6C^Low^CCR2^High^ macrophages, known as monocyte-derived macrophages [9,10]. These are distinct from Ly6C^Low^CCR2^Low^ macrophages deposited in the myocardial tissue during embryonic development [11-13]. Both macrophage populations (Ly6C^Low^CCR2^High^ and Ly6C^Low^CCR2^Low^) contribute to myocardial repair by clearance of dead tissue via efferocytosis and production of pro-reparative and pro-resolving mediators. Macrophage-derived cytokines play an essential role in the proliferation and activation of cardiac fibroblasts (fibroblast-myofibroblast conversion) that deposit collagen at the site of injury. This process of scar formation fulfills the immediate need to preserve the structural integrity of the myocardium, forestalling ventricular rupture [3,14-16]. Thus, acute infiltration of immune cells is necessary for proper infarct healing and preservation of ventricular structure and function after MI. This principle is well supported in experiments interrogating the consequences of systematic neutrophil or monocyte/macrophage depletion on post-MI pathophysiology in pre-clinical models. Results convincingly suggest that a dysregulated immune response after injury leads to impaired infarct healing, ventricular rupture, and exacerbated decline in ventricular performance [2,3,14-16]. Hence, it is only logical that anti-inflammatory/immunomodulatory agents are not a viable therapeutic option for patients with acute MI.

MI often leads to a substantial and irreversible loss of cardiomyocytes (sometimes upwards of 1 billion cells) [2,3,16]. The magnitude of injury, which is governed by a host of factors (i.e., the extent, location, and number of arterial stenoses and time to intervention, among others), is a chief predictor of patient prognoses and progression to heart failure (HF) in the long term. As available therapeutic modalities fail to address the fundamental etiology of this disease (the loss of cardiomyocytes), cell therapy was introduced more than two decades ago as a novel means to resupply the heart with its basic contractile units (cardiomyocytes) and restore cardiac function [17]. Since then, we have learned that the “stem” cells used for therapy neither engraft nor meaningfully contribute to the formation of new cardiomyocytes or vascular structures following their administration *in vivo* [18-23]. Although the injected cells do not contribute to cardiomyocyte regeneration or neovascularization, there is a modest, yet calculable improvement in left ventricular (LV) geometry and function [24,25]. By exclusion, it is thought that administered cells must produce paracrine factors that activate endogenous repair mechanisms—termed “the paracrine signaling hypothesis.” To date, several classes of paracrine factors have been identified, which may help explicate the precise molecular mechanisms underlying the beneficial effects of cardiac cell therapy [19,26-28]. These include cytokines, bioactive lipids, exosomes containing protein, lipids, and various classes of RNA (miRNA, cirRNA, lncRNA) (Figure 1) [20,27,29,30]. Nevertheless, the precise endogenous mechanism(s) responsible for the salutary effects of cell therapy remain largely unknown.

The discovery that bone marrow mesenchymal stromal cells (BM MSCs) are intrinsically immune-privileged and ably suppress T cell proliferation continue to fuel scientific and clinical interests concerning their immuno-regulatory properties. *In vivo* and *in vitro* studies have shown that BM MSCs exert immunomodulatory effects on both innate and adaptive immunity [31,32]. Said studies have laid the foundation for numerous clinical trials employing BM MSCs in chronic inflammatory diseases, including graft versus host disease (GVHD), lupus, Crohn’s disease, and ulcerative colitis [33,34]. Though their immune regulatory actions and therapeutic capabilities have been recognized in numerous disease contexts, the effect of cell therapy on the immune system in heart failure remains poorly described. In our recent study [35], Kang et al. found that cardiac mesenchymal cells (CMC) exert immunomodulatory actions on neutrophils and macrophages. Although overactivation of neutrophils leads to excessive tissue injury, the absence of neutrophils impedes macrophage activation towards a reparative phenotype—inevitably contributing to excess collagen deposition and fibrosis [5]. There is still little understanding concerning the role of neutrophils in the process of myocardial repair. In our study [35], injection of cardiac mesenchymal cells (CMCs) triggered the recruitment of neutrophils to the myocardium. These neutrophils are stratified into two distinct populations: CD206^Neg^, also known as N1, and CD206^Pos^, defined as N2 [36]. In the current study [35], both N1 and N2 neutrophil populations were markedly increased in response to intramyocardial CMC injections. The physiologic importance of this is not clear; however, in light of our previous study showing that CMC administration produces an increase in ventricular function [37] leads us to speculate that myocardial neutrophil infiltration may comprise a mechanism contributing to CMC-mediated cardiac repair. Further, we demonstrate *in vitro* that conditioned medium-derived from CMCs serves as a robust neutrophil chemoattractant, as well as an inhibitor of neutrophil apoptosis [35]. Although neutrophils are important components of myocardial repair, their overactivation could lead to excessive proteolytic enzyme and ROS production—factors that perpetuate injury and contribute to adverse ventricular remodeling and dysfunction [2,14]. It was reported that the secretome of bone marrow mesenchymal cells (BM MSCs) inhibits neutrophil apoptosis and ROS production via IFNβ, GM-CSF, and IL-6 [20]. Thus, it is possible that while cell therapy increases the recruitment of neutrophils to the injured heart, it may also shift their activation towards an anti-inflammatory, pro-reparative phenotype. Given that this purely speculative, further studies are warranted to explore the consequences of cell therapy on neutrophil function/polarization. Such studies may help establish alterations in neutrophil function as an important mechanism of cardiac cell therapy and improve our knowledge regarding fundamental aspects of neutrophil biology and function.

Notwithstanding, it is well recognized that macrophages are imperative for myocardial repair [3,6,14]. In our study [35], we show that CMC administration increases the recruitment of Ly6C^High^ monocytes. Others have shown that MI itself results in the recruitment of Ly6C^High^ monocytes, which follow a CCL2 chemokine gradient [6,9,10,38]. These monocytes give rise to CCR2^Pos^ macrophages that contribute to myocardial repair [7,9,39]. We have found that injection of CMCs enhances the recruitment of monocytes and Ly6C^Low^ macrophages [35]; however, we did not distinguish between CCR2^Pos^ and CCR2^Neg^ populations. In similar studies, Vagnozzi et al. [40] showed that injection of c-kit^Pos^ CPCs or BM MNCs provokes the accumulation of CCR2^Pos^ and CXC3R1^Pos^ myocardial resident macrophages. Furthermore, the authors demonstrate that systemic depletion of macrophages prior to cell injection impairs cell therapy-induced myocardial repair [40]. Thus, this further suggests that donor cell-mediated recruitment of monocytes and their descendant macrophages have salutary effects on myocardial repair, rather than detrimental effects on intrinsic myocardial reparative processes. Thus, these two studies [35,40] suggest that recruitment of monocytes and macrophages could comprise a common mechanism of CMC, CPC, and BM MSC cell-induced myocardial repair. These studies did not interrogate the impact of cell therapy on macrophage function *in vivo*. We show that CMC-derived conditioned medium decreases pro-inflammatory, macrophage polarization in response to LPS and IFNγ, but induces a pro-resolving and pro-reparative program in response to IL-4 and IL-13 [35]; however, we have yet to conduct *in vivo* studies to confirm whether this shift in macrophage polarization is responsible for the beneficial effects of CMC therapy. The primary function of macrophages is to remove necrotic tissue via efferocytosis [2,3,14,15]. We found that CMC conditioned medium enhances Fc receptor-mediated efferocytosis of opsonized latex beads [35]. This is consistent with observations by the Marban laboratory showing cardiosphere-derived cell (CDC) secretome to increase efferocytosis of latex beads by bone marrow macrophages [41,42]. We have confirmed that CMC conditioned medium enhances immune cell efferocytosis of apoptotic cells—findings which have greater physiologic relevance. Removal of dead tissue is an important component of inflammation resolution and repair. First, prolonged exposure of autoantigens provokes an adaptive immune response to generate self-reactive antibodies, and second, signaling from apoptotic cells via TAM (MerTK, Axl, Tyro3) receptors elicits anti-inflammatory and pro-resolving signaling in macrophages [3,14,15]. Thus, one may speculate that increased recruitment and enhanced recognition of apoptotic cells could alter macrophage function to increase repair. However, these studies have not been conducted in *m vivo* experiments.

Although the activation of innate immunity is a hallmark of tissue injury, recently, it was reported that the adaptive immune system is responsive to MI as well [43-46]. After MI, induced by permanent coronary artery ligation, B and T cell levels increase and their numbers peak at approximately 7 days. Detailed flow cytometric analyses demonstrate myocardial B cells display a B220^Pos^CD19^Pos^IgD^Pos^IgM^Low^ mature phenotype [47]. At the same time, there is an infiltration of Foxp3^Pos^ regulatory T cells and also CD4^Pos^ Th cells expressing IFNγ; indicating predominantly Th1-differentiated cells rather than Th2 or Th17 [48,49]. Studies with systemic depletion of B or T cell subpopulations, paired with adoptive transfer of each subpopulation, suggests that adaptive immunity has an important function in the regulation of myocardial repair as well [43,44]. Studies *in vitro* indicate that BM MSCs are potent inhibitors of T cell proliferation, suggesting that cell therapy may also have an impact on adaptive immunity [32-34]. In our study [35], we found that intramyocardial injection of CMCs two days after MI has no effect on B or T cell infiltration 7 days after delivery. Although we concluded that CMCs have no effect on adaptive immunity after MI, we believe it would be prudent to add weight to this pronouncement in rigorously designed follow-up investigations examining additional intervals after cell administration. We measured B and T cells with flow cytometry only at one time point after CMC injection (7 days) [35]. Hence, we cannot exclude the possibility that the donor cells may affect B and T cell infiltration at earlier timepoints. Moreover, in our study we used generic pan B (B220 and CD19) and T (CD4 and CD8) cell markers. Said marker panels are not robust enough to tell us whether there is a shift in B (naïve, plasma cells, memory) and T (Th1, Th2, Th17) cell subsets. It was reported that there is an increase in the proliferation of T cells in heart-draining lymph nodes in response to MI [43,44,49]. Hence, future studies interrogating the effects of cell therapy on T cell activation/proliferation should extend analyses to include additional cardiac regions, such as non-infarct segments/remote myocardium.

Future studies should also address other important questions regarding the immunomodulatory properties of cell therapy. The cardiovascular field would benefit from *in vivo* studies elucidating changes in immune cell function in response to cell therapy. Furthermore, it would be imperative to design interventions aiming to understand how these changes affect myocardial repair after cell therapy. Although the Vagnozzi study [40] demonstrated systemic macrophage depletion with clodronate liposomes impairs the beneficial effects of the cell therapy, authors did not uncover precisely how these immune cells mediate the repair response. Numerous *in vitro* studies have characterized the impact of BM MSC-, CMC-, and CDC-derived secretomes on the phenotypic properties of various immune cell populations (i.e., neutrophils, dendritic cells, monocytes/macrophages, T and B cells [20,35,37,42] (Figure 1); however, there are few robust mechanistic studies demonstrating how changes in immune cell function *in vitro* relate to the reparative potential of donor cells *in vivo*. Furthermore, one of the future challenges would be to identify how injection time after MI impacts the immune system. Currently, it is well known that MI elicits a temporally coordinated immune cell infiltration response, which undergoes dynamic changes over time until the process of replacement fibrosis concludes. The completion of scar formation culminates in the resolution of inflammation [2,3,14]. An important question is whether there is an immunologic, therapeutic window for cell therapy to maximize their reparative response. Growing evidence suggest that chronically after MI there is a gradual accumulation of immune cells that contribute to progressive ventricular remodeling and failure of pump function [39,50]. There are reports suggesting that cell therapy is effective in the chronic phase after MI (~ one month), but there are no studies describing the effect of cell injection on changes in immune cells *in situ* [20]. Perhaps future studies should aim to profile changes in immune cells after cell therapy in the chronic setting of heart failure.

Studies from us [35] and others [40], strongly suggest that cell therapy provides beneficial effects on myocardial structure and function without contribution of the injected cells to regeneration of cardiomyocytes and vasculature. Moreover, increased recruitment of immune cells in response to cell injection suggests that injected cells improve heart function via an immunomodulatory mechanism. In addition to pointing to a potential mechanism of action, our data provide an intervention to study immune-related pathways that may be therapeutically exploited (without the need of donor cell populations or cell-sourced derivatives) to enhance myocardial repair after MI. Un(fortunately), these studies further go against the initial promise of the “stem” cell therapy field by showing that cell administration does not meaningfully contribute to cardiac regeneration. Alternative strategies to increase cardiomyocyte numbers (i.e., induction of proliferative genetic programs in cardiomyocytes, cardiogenic reprogramming of endogenous fibroblasts, etc.) will more likely comprise the solution to the replenishment of cardiac parenchyma after injury. Nevertheless, the recent studies using cell therapy provide valuable information that may help us to understand how the immune system regulates the processes related to replacement fibrosis acutely after MI, but also, how adverse remodeling is regulated in the chronic phase of heart failure.

## Figures and Tables

**Figure 1:**
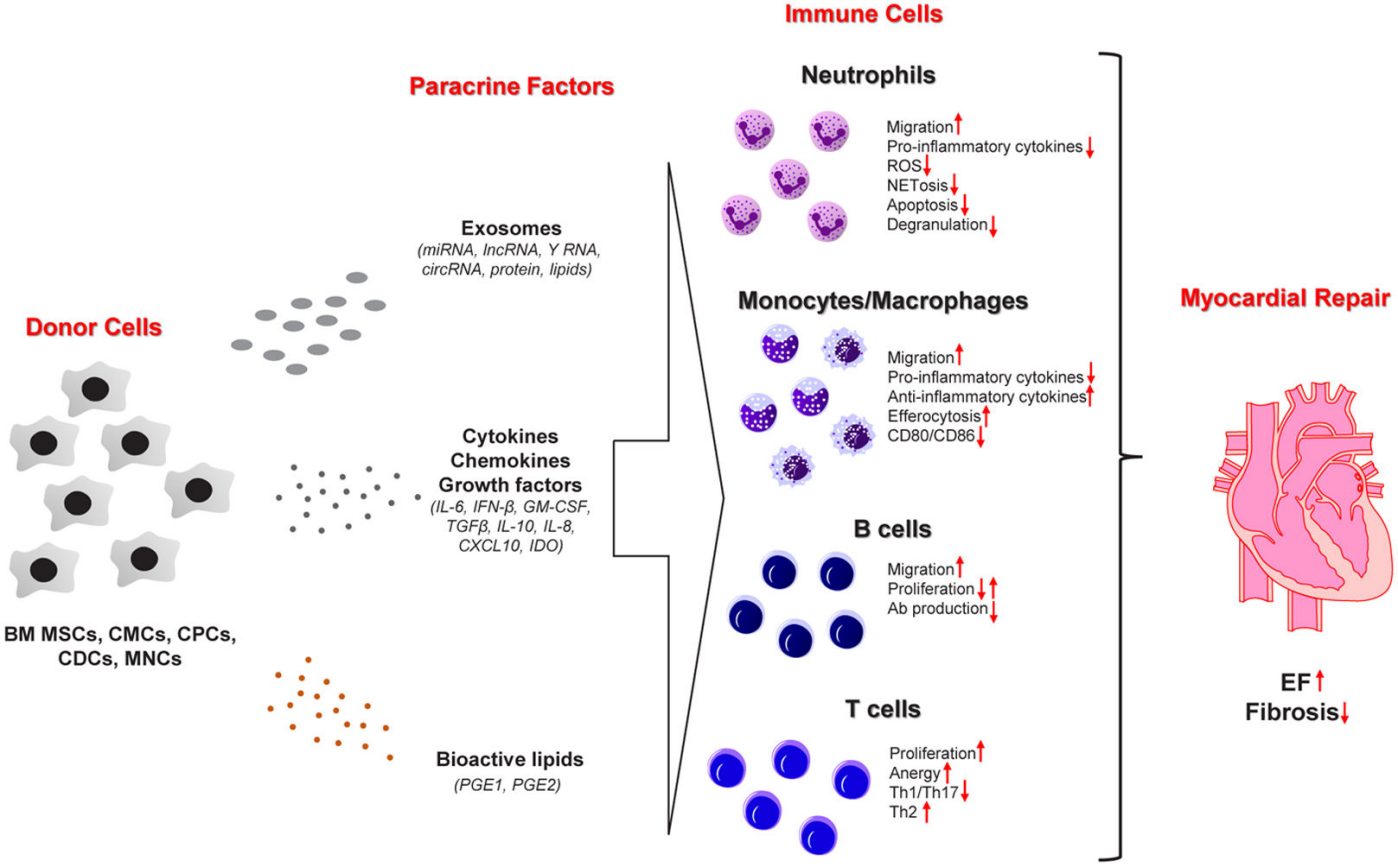
Potential mechanism of myocardial repair after cell therapy via modulation of immune cell function. Donor cells sourced from both cardiac and extra-cardiac tissues produce paracrine factors (exosomes, cytokines, chemokines, growth factors, bioactive lipids) that modify immune cell function/phenotypic properties. Although the effects of donor cell administration on neutrophil, monocyte, and macrophage biology have been reported *in vivo*, their impact on B and T cells remain under explored.
